# Microencapsulation of carvacrol as an efficient tool to fight *Pseudomonas aeruginosa* and *Enterococcus faecalis* biofilms

**DOI:** 10.1371/journal.pone.0270200

**Published:** 2022-07-01

**Authors:** Samah Mechmechani, Adem Gharsallaoui, Alexandre Fadel, Khaled El Omari, Simon Khelissa, Monzer Hamze, Nour-Eddine Chihib

**Affiliations:** 1 University Lille, CNRS, INRAE, Centrale Lille, UMR 8207—UMET—Unité Matériaux et Transformations, Lille, France; 2 Laboratoire Microbiologie Santé et Environnement (LMSE), Faculty of Public Health, Doctoral School of Sciences and Technology, Lebanese University, Tripoli, Lebanon; 3 University Lyon, Université Claude Bernard Lyon 1, CNRS, LAGEPP UMR 5007, Villeurbanne, France; 4 University Lille, CNRS, INRAE, Centrale Lille, Université d’Artois, FR 2638 –IMEC -Institut Michel-Eugene Chevreul, Lille, France; 5 Quality Control Center Laboratories at the Chamber of Commerce, Industry & Agriculture of Tripoli & North Lebanon, Tripoli, Lebanon; Universidade Estadual de Ponta Grossa, BRAZIL

## Abstract

Biofilms are involved in serious problems in medical and food sectors due to their contribution to numerous severe chronic infections and foodborne diseases. The high resistance of biofilms to antimicrobial agents makes their removal as a big challenge. In this study, spray-drying was used to develop microcapsules containing carvacrol, a natural antimicrobial agent, to enhance its activity against *P*. *aeruginosa* and *E*. *faecalis* biofilms. The physicochemical properties and microscopic morphology of the realized capsules and cells were characterized. The minimum inhibitory concentration of encapsulated carvacrol (E-CARV) (1.25 mg mL^-1^) was 4-times lower than that of free carvacrol (F-CARV) (5 mg mL^-1^) against *P*. *aeruginosa*, while it remained the same against *E*. *faecalis* (0.625 mg mL^-1^). E-CARV was able to reduce biofilm below the detection limit for *P*. *aeruginosa* and by 5.5 log CFU ml^-1^ for *E*. *faecalis* after 15 min of treatment. Results also showed that F-CARV and E-CARV destabilize the bacterial cell membrane leading to cell death. These results indicate that carvacrol exhibited a strong antimicrobial effect against both bacterial biofilms. In addition, spray-drying could be used as an effective tool to enhance the antibiofilm activity of carvacrol, while reducing the concentrations required for disinfection of abiotic surfaces.

## Introduction

*Enterococcus faecalis* and *Pseudomonas aeruginosa* are important opportunistic human pathogens that cause major problems in medical and food sectors. In fact, *E*. *faecalis* is a Gram-positive commensal bacterium that is normally associated with human as a member of the gut microflora [1]. However, *E*. *faecalis* is an opportunistic pathogen of considerable clinical importance, particularly as an etiologic agent of healthcare-associated infections [2,3]. In addition, due to their high tolerance to heat and their ability to survive in severe environmental conditions, *E*. *faecalis* can easily persist for long periods of time and contaminate animals’ carcasses and processed products [4–6]. *P*. *aeruginosa* is a Gram-negative bacterium that can provoke several healthcare-associated infections, even fatal infections in immunosuppressed patients [7]. Furthermore, the involvement of *P*. *aeruginosa* in foodborne infections and food spoilage is also reported [8].

In natural and artificial ecosystems, bacteria may adhere to surfaces and form a complex three-dimensional structure called biofilm [9]. Bacterial cells in biofilm are enclosed in an extracellular polymeric substances (EPS) of proteins, polysaccharides, and nucleic acids that can serve as a barrier providing a resistance to various hostile conditions such as antibiotics, disinfectants, and other sanitizing conditions, and preventing thus their penetration into the deeper layers of biofilms. Therefore, those bacterial cells are more resistant to antibacterial agents or antibiotics than planktonic cells [10]. *E*. *faecalis* is frequently isolated from biofilms formed on the surfaces of various indwelling medical devices related to chronic infections [11]. In addition, Enterococcal biofilms formed in food processing environments are very difficult to eradicate, making them one of the most prevalent opportunistic pathogens and spoilage bacteria in meat products [6,12–14]. *P*. *aeruginosa* also effectively colonizes various surfaces, including medical equipment (implants, urinary catheters, contact lenses, etc.) causing many chronic infections, and food industry equipment (tanks, mixing tanks and pipes) causing food spoilage [7,15].

Plant-derived essential oils are natural antimicrobial agents with effective antimicrobial activity against bacteria, fungi and viruses. Carvacrol [2-Methyl-5-(1-methylethyl) phenol], a volatile monoterpene, is a major component of many essential oils of the Labiatae family plants, including *Thymus*, *Origanum*, *Satureja*, and *Coridothymus* species. Carvacrol is classified as generally recognized as safe (GRAS) by the Food and Drug Administration for its uses in food as preservative and food flavoring ingredient [16,17]. Carvacrol is known for its broad antimicrobial activity against foodborne or pathogenic microorganisms, including drug-resistant bacteria [18,19]. However, the application of carvacrol is limited due to its low stability, poor water solubility and high volatility [20].

Microencapsulation techniques have been widely used in the pharmaceutical and food industries to control the release of active molecules, improve the stability of formulations and mask flavors [21,22]. In addition, microencapsulation may reduce the amounts of biocides used and thus decreases their negative environmental impacts. This technique can also impede the interactions of antimicrobials with biofilm EPS matrix, which can lead to repulsion or retention of the biocide and prevent their interactions with microbial cells, thus allowing for deep layer biofilm disinfection. Therefore, the microencapsulation of natural terpenes could also be a promising method to surmount their water immiscibility, volatility and cytotoxicity. Many microencapsulation techniques are commonly used, including spray-drying, extrusion, and coacervation [23].

In the current study, feed emulsions were first prepared using sodium caseinate as emulsifier and maltodextrins as drying matrix. Emulsions were then spray-dried to obtain dry carvacrol microcapsules. The antimicrobial activity of free and microencapsulated carvacrol was assessed against *P*. *aeruginosa* and *E*. *faecalis* biofilms performed on stainless steel. The main objective was to improve the efficiency of this antimicrobial agent against pathogenic bacterial biofilms through its spray-dried formulation, while reducing the amount used.

## Material and methods

### Growth conditions and cell suspension preparation

*P*. *aeruginosa* (CIP 103467) and *E*. *faecalis* (isolated from French cheese) were used in this study. The strains were maintained in tryptic soy broth (TSB; Biokar Diagnostics, France) supplemented with 40% (v/v) glycerol at -80°C. Prior to use, bacteria were pre-cultivated by inoculating 100 μL of the frozen strain cultures into 5 mL of TSB medium and incubating for 24 h at 37°C. Then 100 μL of the pre-culture were used to inoculate 50 mL of TSB medium in 500 mL sterile flasks, and incubated for 16 h at 37°C under shaking conditions at 160 rpm to prepare the culture. Cells were pelleted by centrifugation for 5 min at 5000 × *g*. Then, bacteria were washed twice with 20 ml of potassium phosphate buffer (PPB; 100 mM, pH 7) and finally re-suspended in PPB. The cells were dispersed by sonication at 37 kHz (Elmasonic S60H, Elma®) for 5 min at 20°C.

### Antimicrobial agents

Carvacrol (98% purity) was purchased from Sigma-Aldrich (St. Louis, MO, USA). Dimethyl sulfoxide (DMSO; Sigma-Aldrich, France) with a final concentration of 2% (v/v) was used to prepare F-CARV solution, while E-CARV did not require DMSO since microcapsule already contain emulsifier that solubilize carvacrol.

### Microencapsulation of carvacrol

Carvacrol emulsions were prepared by dissolving sodium caseinate in water while shaking at room temperature until complete hydration. The pH was then adjusted to 7 using HCl (0.1 or 1 mol L^-1^) or NaOH (0.1 or 1 mol L ^-1^). Each emulsion was then mixed with a maltodextrin DE 19 solution (50% w/v) to obtain a feed emulsion with the following composition: carvacrol (1%), sodium caseinate (0.5%), and maltodextrins (20%). The pH of the feed emulsions was readjusted to 7 before analysis.

Feed emulsions were shaken for 30 minutes and subsequently spray-dried by using a lab-scale device equipped with a 0.5 nm nozzle atomizer (Mini Spray-Dryer Buchi B-290, Switzerland). The drying process operational conditions were as follow: outlet air temperature 80 ± 5°C, inlet air temperature 180 ± 2°C, and feed flow rate 0.5 L h^-1^. Powders were collected in separate sealed vessels after spray-drying and stored at 4°C until testing. Reconstituted suspensions were then prepared by scattering weighted quantities (the same amount of dry matter as before spray-drying) of spray-dried powders in water and shaking for 1 h.

### Zeta potential measurement

The electrical charge (ζ-potential) of the emulsions before and after spray-drying and reconstitution was determined using a Zetasizer Nano ZS90 (Malvern Instruments, Malvern, UK). If needed, samples were diluted in water with the appropriate pH. For each test, at least three repetitions were performed. The average of ζ-potential (ZP) values was obtained from the instrument.

### Scanning electron microscopy (SEM) analysis of microcapsules and cells

The internal and external structures of dried carvacrol microcapsules were examined using SEM (JEOL-JSM-7800FLV, Japan). To observe the external structure, a dry powder layer was simply attached to a sample holder with a double-sided adhesive (Agar Scientific Oxford). To study the internal structure, powders containing carvacrol microcapsules were smashed by moving a razor blade perpendicularly through a layer of microcapsules. The morphology of treated biofilm cells was also investigated using SEM. Briefly, bacterial cells were recovered after biofilm treatment by scraping the surface, aspirating and expelling with 6 mL of ultrapure water at least 10 times. Recovered cell suspensions were vortexed for 30 s, followed by sonication (5 min, 37 kHz) then diluted tenfold in Tryptone Salt broth (TS; Biokar Diagnostics, France). Samples (1 mL) were filtered using a polycarbonate membrane filter of 0.2 μm pore size (Schleicher & Schuell, Dassel, Germany) then fixed with cacodylate buffer 0.1 M, pH 7.0 (sodium cacodylate trihydrate (CH3)_2_AsO_2_Na.3H_2_O) containing 2% glutaraldehyde, at 4°C. Fixed bacterial cell samples were dehydrated in an upward series of ethanol (50, 70, 95, and 2×100% (v/v) ethanol) for 10 min at each concentration and critical point dried. Fixed bacterial cell or dried microcapsules samples were covered with a thin carbon film before scanning by SEM. Microscopy was performed at 3 kV.

### Antimicrobial activity of carvacrol against planktonic cells

#### Determination of the minimal inhibitory of planktonic cells

The minimum inhibitory concentration (MIC) of F-CARV and E-CARV was determined using Mueller-Hinton Broth (MHB; Biokar Diagnostics, Pantin, France) by a microdilution growth inhibition assay using a Bioscreen C (Labsystems, Helsinki, Finland) that measures turbidity by vertical photometry. Briefly, 100 μL of double serial dilutions of F-CARV and E-CARV (from 10 to 0.156 mg mL^-1^) were carried out in the 96-well microdilution plates. Subsequently, 100 μL of *P*. *aeruginosa* (CIP 103467) and *E*. *faecalis* suspensions (10^6^ CFU mL^-1^) were added. Bacteria were not added to negative control, while in positive controls, DMSO without antimicrobials was used for F-CARV and the same control without DMSO was used for E-CARV. The plates were incubated in Bioscreen C at 37°C with a continual shaking and the optical density (OD600 nm) was read every 2 h for 24 h. MIC value was defined as the lowest concentration of the antimicrobial agent that prevents the bacteria from obvious growth in the microdilution wells after incubation.

#### Time kill assay

The time kill test was used to study the bactericidal effects of the antimicrobial agents. The experiment was performed according to Isenberg (2004) with some modifications. Briefly, bacteria were overnight cultured and transferred at MIC values to MHB, supplemented with F-CARV and E-CARV, to obtain a final inoculum of 10^6^ CFU mL^-1^. Control containing DMSO was used for F-CARV and without DMSO for E-CARV. Then, bacteria were incubated at 37°C with shaking. At the selected time, 100 μL were taken, diluted serially, and plated on Mueller Hinton agar (MHA, Difco Pont-de-Claix, France). Plates were incubated at 37°C for 24 h and the colony forming units (CFU) were enumerated. Tests were replicated three times.

### Carvacrol-induced potassium ion leakage

Planktonic *P*. *aeruginosa* cells grown at 37°C were concentrated to 10^10^ CFU mL^-1^ (5 000 × *g*, 15 min, 20°C). Tenfold dilutions of the concentrated bacterial suspensions were prepared in 50 mM morpholinopropane sulfonic buffer (MOPS; Fisher scientific, Belgium) containing F-CARV and E-CARV (at the MICs). K^+^ concentrations were measured at the time 0, 5 and 10 min in a tenfold dilution of the concentrated bacterial suspension filtrate (0.2 μm, Sartorius™ Minisart™ NML Syringe Filters, France) before contact with the antimicrobial solutions. After the exposure of the bacterial suspension cells to the F-CARV and E-CARV solutions, samples (4 mL) were filter sterilized at 0.5, 1, 1.5, 2, 2.5, 3, 3.5, 4, 4.5, 5, 10, 15, 20, 30 and 40 min. MOPS buffer with DMSO was used as control for F-CARV and without DMSO for E-CARV. The concentration of K^+^ in the filtrate samples was measured using a Varian SpectrAA 55/B atomic absorption spectrometer in flame emission mode (slit 0.7 nm high; wavelength 766.5 nm; air-acetylene flame).

### Assessment of the mechanism of activity of carvacrol on *Pseudomonas aeruginosa* GFP

The experiment was performed according to Khelissa et al. [24] with some modifications. After overnight culture of *P*. *aeruginosa* GFP (ATCC ®10145GFP™) in TSB supplemented with 100 mg mL^-1^ ampicillin, cells were harvested by centrifugation (5 000 × *g*, 5 min, 20°C), washed twice with HEPES buffer (5 mM, pH 7.2), and resuspended in HEPES buffer to obtain a concentrated inoculum (10^10^ CFU mL^-1^). Tenfold dilutions of this concentrated inoculum were prepared in HEPES buffer and sterilized by filtration (Sartorius ™ Minisart™ NML 0.2 μm Syringe Filters, France) at 0, 5, and 10 min prior to exposure of the cells to carvacrol. These filtrates were used to determine the extracellular fluorescence intensity of GFP before antibacterial treatments. In order to expose cells to carvacrol, tenfold dilutions of the concentrated inoculum were prepared in HEPES buffer supplemented with F-CARV and E-CARV (at the MICs). The samples were sterilized by filtration at 5, 10, 15, 20, 30 and 40 min after exposure of the cells to the antimicrobial solutions. HEPES buffer with DMSO was used as control for F-CARV and without DMSO for E-CARV. To quantify GFP fluorescence, 200 μL of the filter samples were transferred to a 96-well microplate and measured by BioTek fluorescence spectrophotometer (BioTek Instruments SAS, France) with excitation at 485 nm and emission at 510 nm. The ratio of fluorescence intensity of the samples was plotted against contact time. The results represent the mean of three independent experiments.

In order to demonstrate that the increase in extracellular GFP fluorescence intensity indicates cell membrane damage and subsequent cell death, the percentage of viable cells in the treated suspension was calculated after staining with LIVE/DEAD® BacLight kit (Invitrogen Molecular Probes, USA). Briefly, after treatment of the suspension with the antimicrobial solutions (at very low concentrations to monitor the effect of carvacrol over time), 10^7^ CFU mL^-1^ of the suspension were filtered with polycarbonate filter (0.2 μm-pore-size, Millipore, France) at 5, 10, 15, 20, 30 and 40 min and then stained in the dark for 10 min. Stained cells were rinsed with 1 mL of distilled water, and filters were kept in the dark to air-dry, then viable cells (green) and dead cells (red) were enumerated using epifluorescence microscope (Olympus BX43, Germany) over 50 microscopic fields. The percentage of viable cells was calculated using the following formula: (number of green cells × 100 / number of total cells (red and green cells)). Results represent the average of three independent experiments.

### Anti-Biofilm assessment using free and encapsulated carvacrol

#### Preparation of stainless steel slides

Stainless steel (SS) slides (304L, Equinox, France) of 41 mm diameter and 1 mm thickness were used for this study. Slides were immersed overnight in 95% ethanol (Fluka, Sigma-Aldrich, France), then washed with distilled water. The rinsed slides were immersed again in 1% DDM ECO detergent (ANIOS, France) for 15 min at 20°C. The slides were afterwards washed vigorously with distilled water 5 times for 1 min, followed by three ultra-pure water washes (Milli-Q® Academic, Millipore, France) to completely remove detergent residues. Finally, SS slides were air-dried before sterilization by autoclaving at 121°C for 20 min. The sterile slides were placed in a static biofilm reactor, called *NEC biofilm System*, for biofilm deposition. This system as previously described by Abdallah et al. [25], is composed of several assembled SS parts and a rubber O-ring. Briefly, the SS lower part forms the circular base of the system, and the O-ring placed on the upper plate side is used to tightly match the SS slide. The SS cylinder with two holes is used to form a well for biofilm formation with oxygen supply. The clamp is used for sealing and the metal cover is used to maintain the sterility of the closed system. All parts were autoclaved at 121°C for 20 min.

#### Biofilm formation assay

After placing sterile slides in the *NEC biofilm system*, 3 mL of bacterial suspension (10^7^ CFU mL^- 1^) of *P*. *aeruginosa* and *E*. *faecalis* were deposited on the SS slides in each reactor and incubated at 20°C for 60 min aiming the adhesion of bacterial cells to surfaces. Beyond this period, the 3 mL were discarded and the slides were carefully washed twice with 5 mL of PPB to remove non-adhered cells. Then, each slide with adhered cells was covered by 5 mL of TSB and the sealed systems were incubated for 24 h at 37°C. After incubation, the old TSB medium was discarded and biofilms covering the SS slides were rinsed twice with 5 mL PPB to eliminate planktonic cells. Rinsed slides were then used for the antibiofilm treatments, the quantification of biofilm biomass and the epifluorescence microscopy analysis.

#### Antibiofilm assay

In order to treat biofilms with antimicrobial agents, rinsed slides were placed horizontally in 3 mL of F-CARV or E-CARV solution at ½ MIC and MIC concentrations of F- CARV for each strain (5 mg mL^-1^ and 2.5 mg mL^-1^ for *P*. *aeruginosa*, 0.625 mg mL^-1^ and 0.312 mg mL^-1^ for *E*. *faecalis*), and treated for 1, 5 and 15 min at 20°C. Control 1 was performed by submerging the slides in 3 mL of TS; in control 2, the slides were immerged in 3mL of TS broth containing DMSO. After treatment, slides were removed from the disinfectant solution and soaked into 5 mL of neutralizing solution (containing a combination of Saponin (30 g L^-1^), Sodium Thiosulphate (5 g L^-1^), Tween 80 (30 g L^-1^), L-Histidin (1 g L^-1^), Lecithin (30 g L^-1^), and TS broth (9.5 g L^-1^) to block the antibacterial action [26]. For the epifluorescence microscopy analysis, the slides were transferred to petri dishes and the biocide action was stopped by applying 3 mL of neutralizing solution on the top side.

For the quantification of biofilm biomass, slides were placed into 20 mL TS broth using a sterile 100 mL pot. Attached cells were detached by vortexing for 30 s, sonication for 5 min (37 kHz, 5 min, 25°C) (Elmasonic S60H, Elma, Germany), followed by vortexing again for 30 s. Thereafter, serial dilutions were prepared in TS broth and plated onto Tryptic Soy Agar (TSA; Biokar Diagnostics, France) plates. The number of cells was counted on the plates after incubation for 24 h at 37°C and the results were presented as log CFU mL^-1^. Results represent the average of three independent experiments.

### Epifluorescence microscopy analysis

Treated biofilms were stained with the LIVE/DEAD® BacLight kit (Invitrogen Molecular Probes, USA) for 15 min in the dark, according to the manufacturer’s instructions. After staining sessile cells, slides were thoroughly rinsed with distilled water and then kept in the dark to air dry. Thereafter, epifluorescence microscopy observations were performed using an epifluorescence microscope (Olympus BX43, Germany). Green cells were designated as viable and red cells were considered as non-viable.

### Statistical analysis

Each experiment was repeated at least three times. Statistical significance was determined by GraphPad Prism 9.0 software using one-way ANOVA (Tukey’s multiple comparisons test). Values of *p < 0*.*05* were considered statistically significant.

## Results

### Zeta potential of carvacrol droplets

During the preparation of the carvacrol microcapsules, the pH of the emulsion was adjusted to 7. The measurement of the zeta potential of the dispersed droplets gave an average value of -24.39 mV. This negative value is explained by the fact that sodium caseinate was used as an emulsifier at a pH higher than the isoelectric point (pHi~4.5). Indeed, proteins show an overall negative charge when the pH exceeds the pHi because of the ionization of the carboxyl groups (COOH—> COO^-^). With the aim of studying the effect of spray-drying process (thermomechanical treatment combining shearing and heating), the zeta potential was measured after reconstitution of the powder. The average value obtained was -23.57 mV, which proves that the presence of maltodextrins as a drying matrix makes it possible to preserve the structure of the protein interfacial membrane which surrounds the carvacrol droplets.

### SEM morphology of spray-dried microcapsules

**Fig 1:**
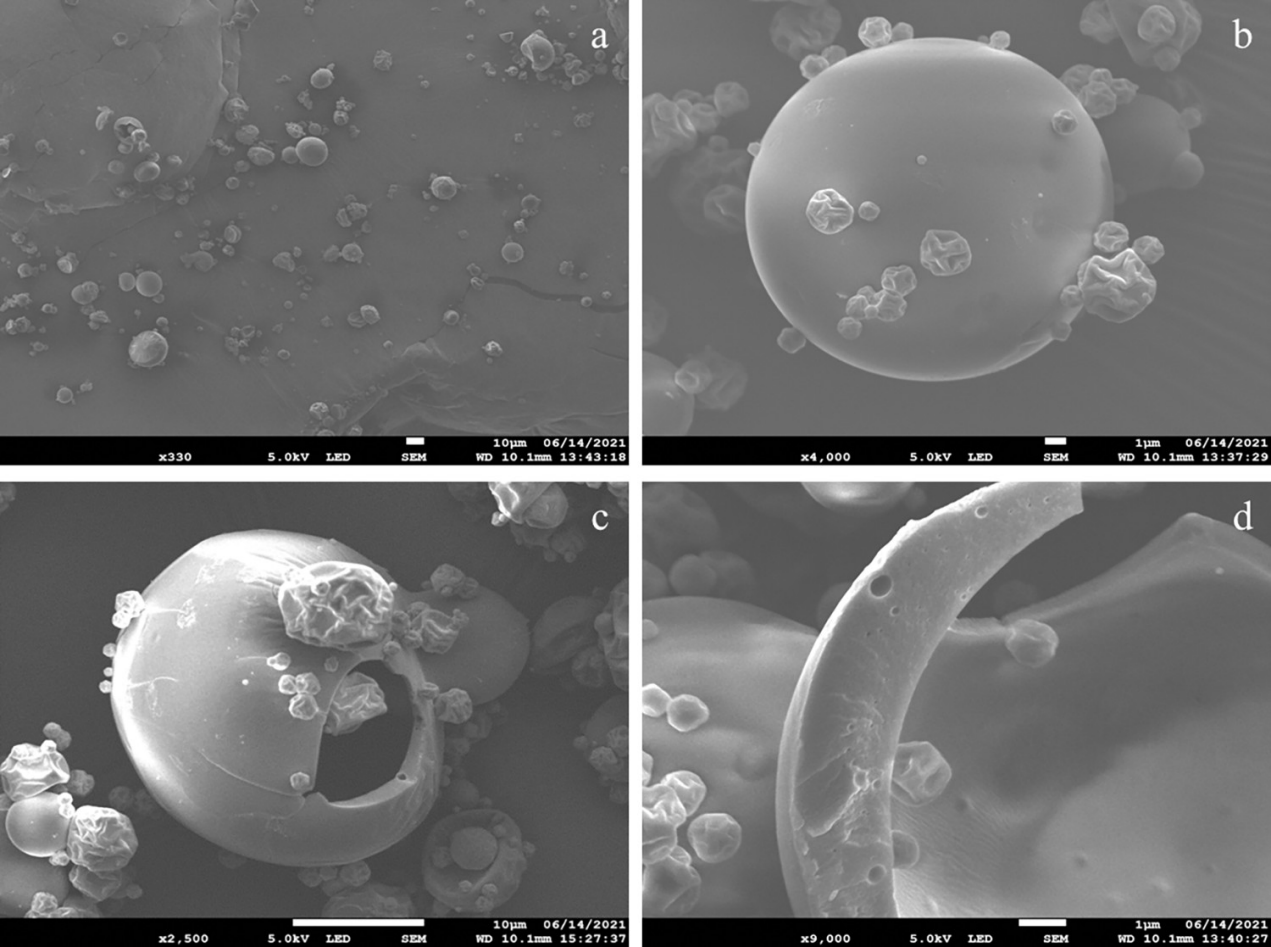
SEM images of carvacrol spray-dried microcapsules (E-CARV). Overview of a multitude microcapsules (a) (330×); Outer morphology of E-CARV microcapsules (b) (4000×); Inner structure of E-CARV microcapsules (c) (2500×) and (d) (9000×).

Fig 1 shows the scanning electron micrographs of microcapsules obtained by spray-drying emulsions of carvacrol in the presence of maltodextrins DE19 and sodium caseinate. The microcapsules were spherical and well-separated with non-uniform diameters (Fig 1A), a blunted shape, and generally bumpy surfaces, with the presence of some small shrinked particles having a rough surface (Fig 1B). The internal structure observations of the formed microcapsules showed the presence of a boundary air bubble (called void) in the center (Fig 1C). In addition, the wall matrix of these microcapsules appeared thickened and typically hollow, with an obvious encapsulated core material retained within (Fig 1D).

### Determination of the Minimal Inhibitory Concentrations (MIC) of free and encapsulated carvacrol

The MIC values of F-CARV were determined and compared with those of E-CARV against *P*. *aeruginosa* and *E*. *faecalis* strains. Results showed that the use of DMSO, at a final concentration of 2% (v/v), to improve the miscibility of carvacrol in water, did not affect the growth of the strains studied. This suggests that at this concentration DMSO is not toxic to the bacteria cells studied. The MIC values of F-CARV and E-CARV against *E*. *faecalis* were similar, corresponding to 0.625 mg mL^-1^ (Table 1). However, for *P*. *aeruginosa*, the MIC value of E-CARV (5 mg mL^-1^) was 4 times lower than that of F-CARV (1.25 mg mL^-1^) (Table 1).

**Table 1:** Minimal inhibitory concentrations of F-CARV and E-CARV against *P*. *aeruginosa* and *E*. *faecalis* (in mg mL^-1^).

Bacterial strains	F-CARV	E-CARV
***Pseudomonas aeruginosa***	5	1.25
***Enterococcus faecalis***	0.625	0.625

### Carvacrol time killing determination

The assessment of the time required to kill over than 99% of the total bacterial population of 10^6^ log CFU mL^-1^ was performed by time kill assay. The control cell suspension of both *P*. *aeruginosa* and *E*. *faecalis* showed a bacterial population of approximatively 6 log CFU mL^-1^ (*p <0*.*05*). When the target bacterial cells were exposed to the MIC of both F-CARV and E-CARV a rapid killing of over than 99% of the total *P*. *aeruginosa* and *E*. *faecalis* populations was observed during the first 1 min and 5 min of exposure, respectively.

### Assessment of the anti-biofilm activity of free and encapsulated carvacrol

The antibiofilm effect of F-CARV and E-CARV was performed on *P*. *aeruginosa* and *E*. *faecalis* biofilms grown on SS for 24 h at 37°C. This experiment aimed to assess the effect of contact time and carvacrol concentrations on the biofilm removal, as well as the difference in antibiofilm efficiency between F-CARV and E-CARV.

**Fig 2:**
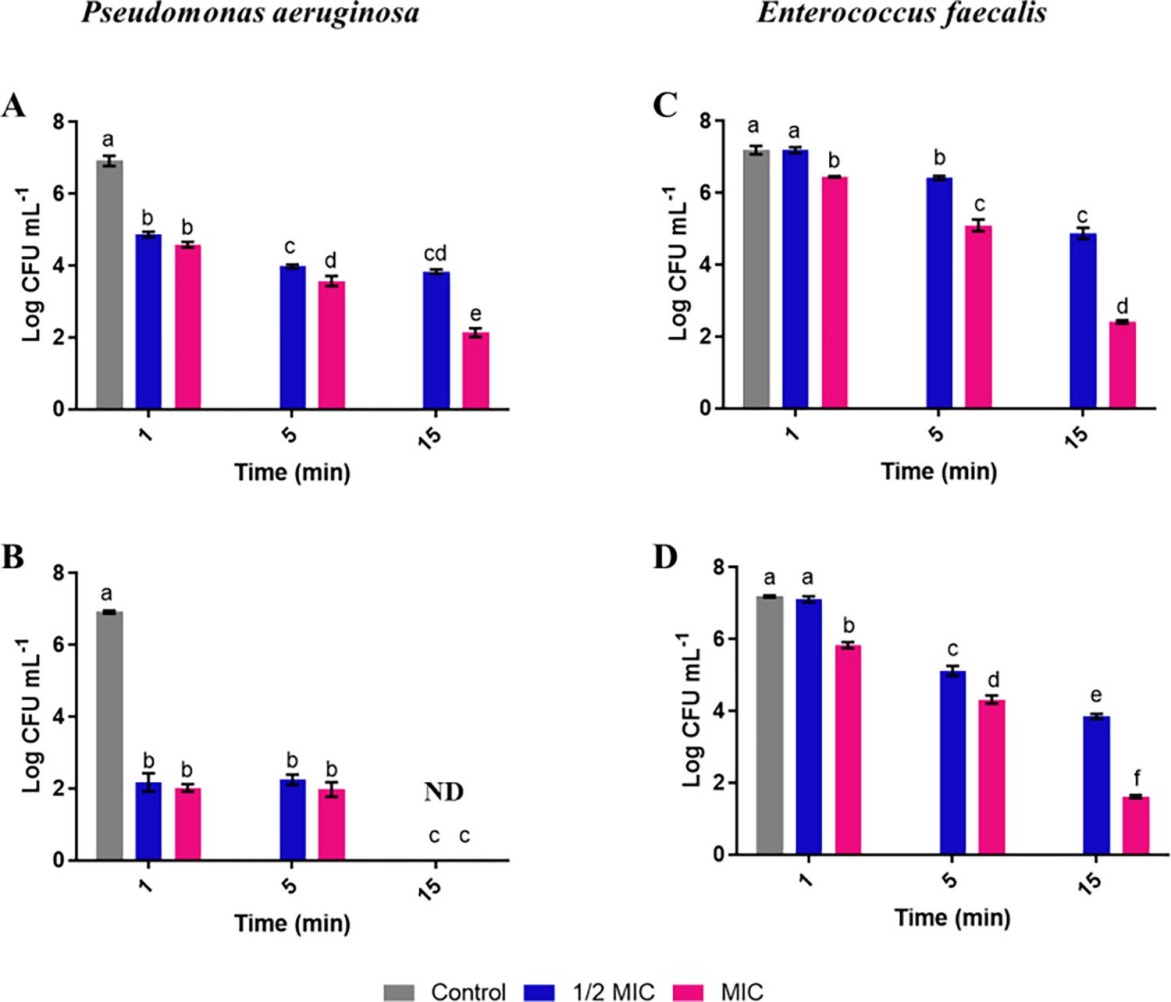
Effect of F-CARV (A, C) and E-CARV (B, D) on the total biofilm biomass of *P*. *aeruginosa* and *E*. *faecalis*. Results are expressed as the mean (± SD) of three independent experiments. Control represents biofilms treated with tryptone salt broth with DMSO for F-CARV and without DMSO for E-CARV. ND: Non detectable bacteria. Different letters at the top of the error bars (a, b, c, …) represent significant differences (*p < 0*.*05*); the same letters represent non-significant differences (*p > 0*.*05*).

*P*. *aeruginosa* biofilms presented a bacterial biomass of approximatively 7 log CFU mL^-1^. The biomass reduction obtained after treatment by the F- CARV with ½ MIC value for 1 min was 2 log CFU mL^-1^ (*p <0*.*05*) (Fig 2A). This reduction was increased to 3 log CFU mL^-1^ (*p <0*.*05*) after 5 min of treatment as well as after 15 min (Fig 2A). Using the MIC value, the reduction in biofilm biomass after 15 min of treatment by F-CARV reached 4.8 log CFU mL^-1^ (*p <0*.*05*) (Fig 2A). However, the biomass reduction obtained with E-CARV was more effective compared to F-CARV using ½ MIC and MIC concentration (Table 2). Results showed that the reduction was approximately similar after 1 min and 5 min treatment using ½ MIC and MIC concentrations (≈ 5 log CFU mL^-1^) (*p <0*.*05*) (Fig 2B). However, the results of Fig 2B showed that the treatment for 15 min using ½ MIC and MIC concentrations was the most effective treatment, since there were no colonies detected suggesting that more than 99% of biofilm cells were killed.

**Table 2:** Statistical analysis of the results of *P*. *aeruginosa* biofilm treatment with F-CARV and E-CARV.

	½ MIC			MIC		
Time (min)	1	5	15	1	5	15
**F-CARV**	A	A	A	A	A	A
**E-CARV**	B	B	B	B	B	B

Different letters (A and B) between F-CARV and E-CARV treatment represent significant differences (*p < 0*.*05*); the same letters represent non-significant differences (*p > 0*.*05*).

For *E*. *faecalis*, the biofilm presented a bacterial biomass of approximatively 7 log CFU mL^-1^. After 1 and 5 min of treatment with ½ MIC value of F-CARV, there were no significant biofilm biomass reductions, whereas after 15 min of treatment, the biofilm biomass was reduced by 2.3 log CFU mL^-1^ (*p <0*.*05*) (Fig 2C). Using MIC value, the antibacterial effect of F-CARV increased with treatment time and resulted in a reduction of 2.1 and 4.7 log CFU mL^-1^ (*p <0*.*05*), after 5 and 15 min, respectively (Fig 2C). However, when *E*. *faecalis* biofilms were exposed to E-CARV, reduction of biofilm biomass was more significant than F-CARV using ½ MIC and MIC concentrations, except after 1 min of treatment with ½ MIC concentration where there was no reduction in biofilm biomass compared to the control (Table 3). The reductions obtained after 5 and 15 min of treatment using ½ MIC concentrations were 2 and 3.3 log CFU mL^-1^ (*p <0*.*05*), respectively (Fig 2D). This reduction was more significant using the MIC concentration and resulted in a reduction of 1.3, 4.3 and 5.5 log CFU mL^-1^ (*p <0*.*05*), after 1, 5 and 15 min of treatment, respectively (Fig 2D).

**Table 3:** Statistical analysis of the results of *E*. *faecalis* biofilm treatment with F-CARV and E-CARV.

	½ MIC			MIC		
Time (min)	1	5	15	1	5	15
**F-CARV**	A	A	A	A	A	A
**E-CARV**	A	B	B	B	B	B

Different letters (A and B) between F-CARV and E-CARV treatment represent significant differences (*p < 0*.*05*); the same letters represent non-significant differences (*p > 0*.*05*).

### Effect of free and encapsulated carvacrol on biofilm bacterial cells morphology

**Fig 3:**
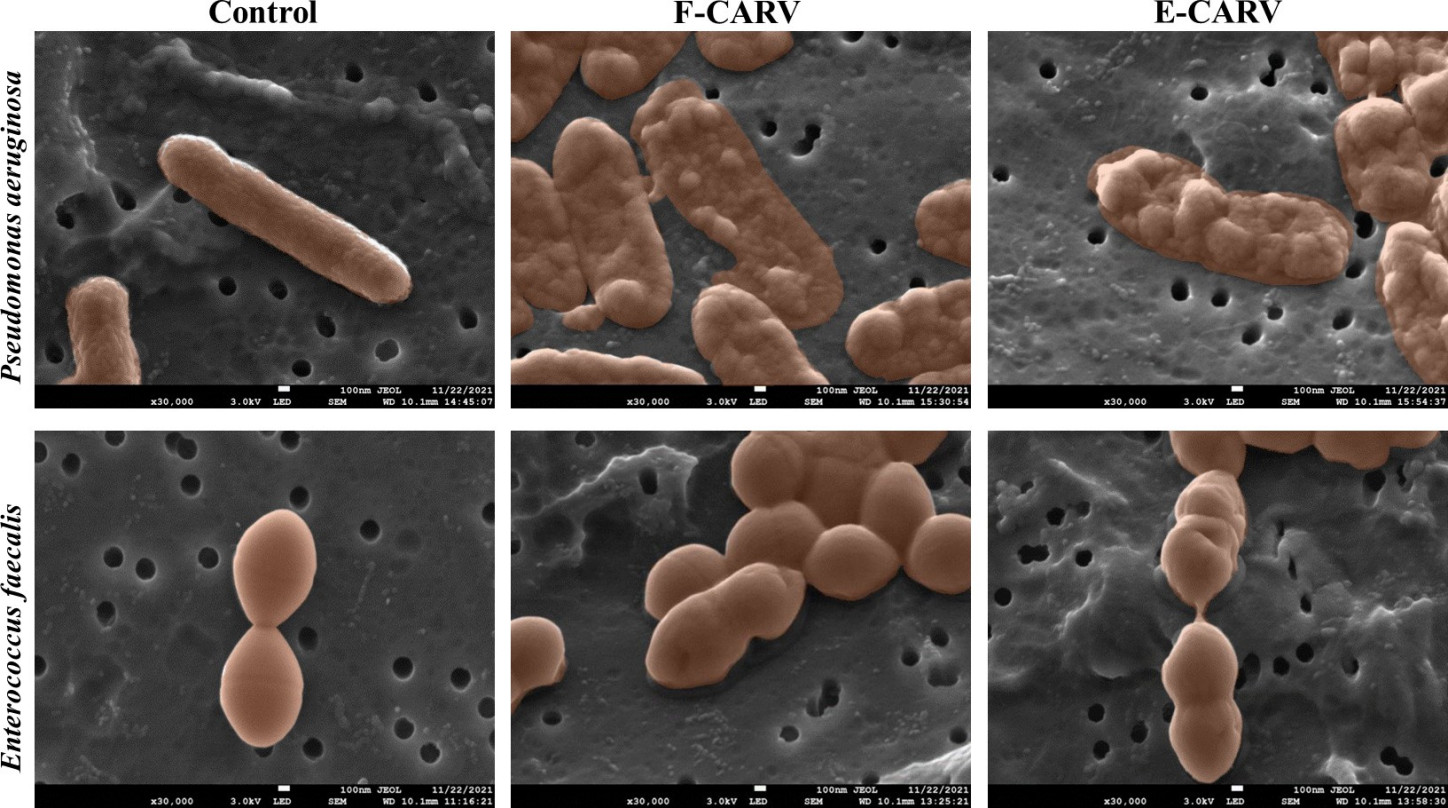
SEM micrographs of *P*. *aeruginosa* and *E*. *faecalis* biofilm cells after treatment with F-CARV and E-CARV. The control represents biofilm cells treated with tryptone salt containing DMSO for F-CARV and without DMSO for E-CARV.

To investigate the effect of F-CARV and E-CARV on bacterial cell morphology, the biofilm cells of *P*. *aeruginosa* and *E*. *faecalis* were analyzed using SEM. The electromicrographs obtained are shown in Fig 3. The TS-treated biofilm (negative control) of both bacterial strains showed intact cells with a normal and regular structure. However, cells treated with F-CARV and E-CARV at the MICs showed different morphological changes and deformation of bacterial cell structures. For *P*. *aeruginosa*, a complete cell shrinkage and deflation was observed after F-CARV and E-CARV treatment, providing evidence of membrane destruction and leakage of the intercellular pool. Moreover, *E*. *faecalis* cells treated with both carvacrol treatments appeared irreversibly damaged, deformed, and had holes in their cell walls.

### Impact of antimicrobial treatment on biofilm structure and cell viability

**Fig 4:**
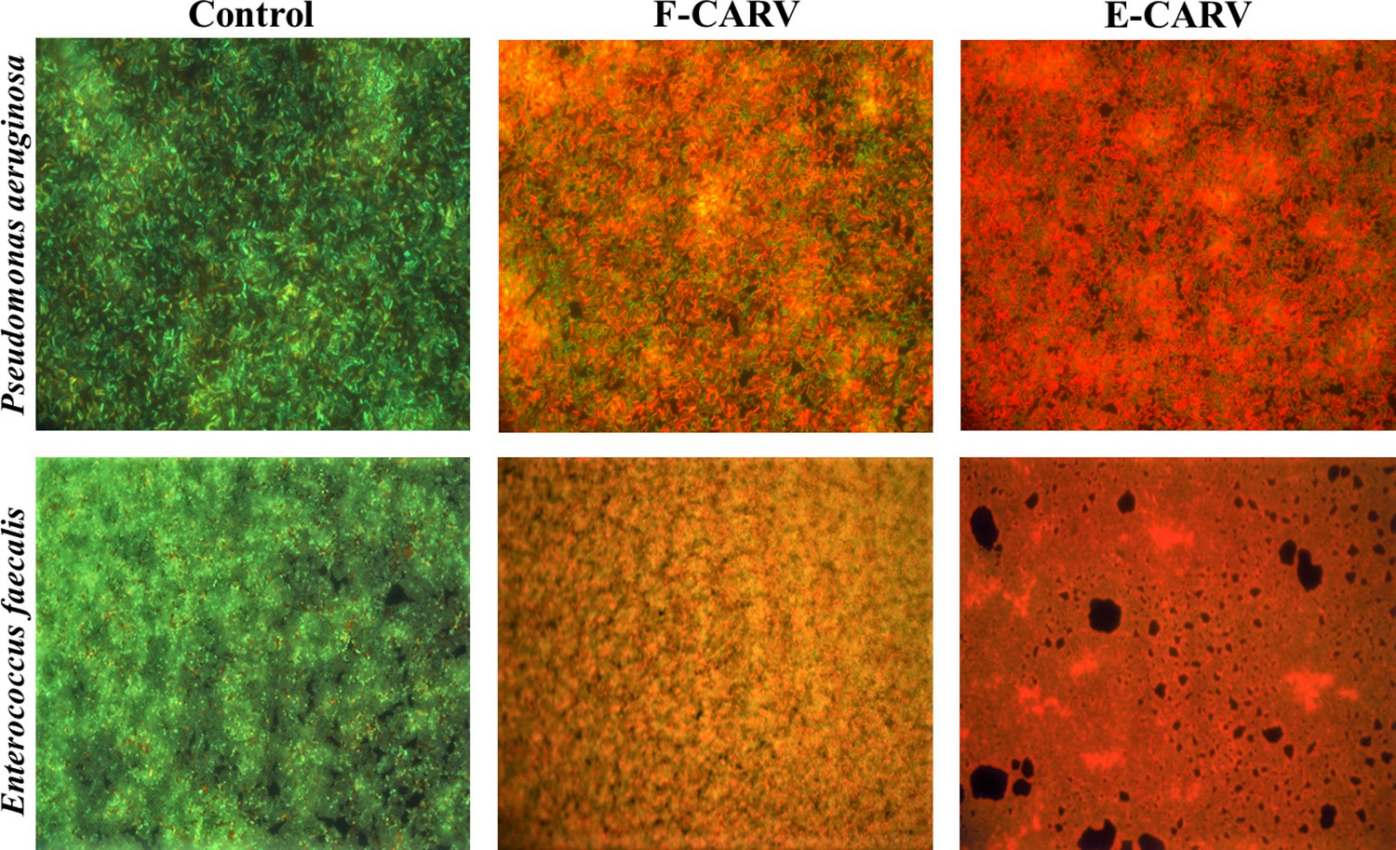
Fluorescence microscopic images of *P*. *aeruginosa* (A) and *E*. *faecalis* (B) biofilms after treatment with F-CARV and E-CARV. Cells were visualized after staining with SYTO-9 and propidium iodide. Green cells represent living bacteria and red fluorescence represents dead bacteria. The control represents biofilm treated with tryptone salt broth.

The 24-hour biofilms of *P*. *aeruginosa* and *E*. *faecalis* were stained with SYTO9 and propidium iodure (PI) after treatment with F-CARV and E-CARV at the MICs and observed by epifluorescence microscopy (Fig 4). For both bacteria, results showed that the TS-treated control exhibited a dense biomass of viable biofilms mainly stained with SYTO9 (green bacteria) with very few dead bacteria stained with PI (red bacteria). After treatment with F-CARV and E-CARV, results showed a representative decrease in SYTO9 staining and an increase in PI staining of the remaining biofilm. Furthermore, treatment with E-CARV induced more cell death than F-CARV, presented by an increase in the PI-stained cell layer.

### Effect of free and encapsulated carvacrol treatment on *Pseudomonas aeruginosa* and *Enterococcus faecalis* cytoplasmic cell membrane permeability

#### Effect of carvacrol on potassium gradient in *Pseudomonas aeruginosa* and *Enterococcus faecalis* cells

**Fig 5:**
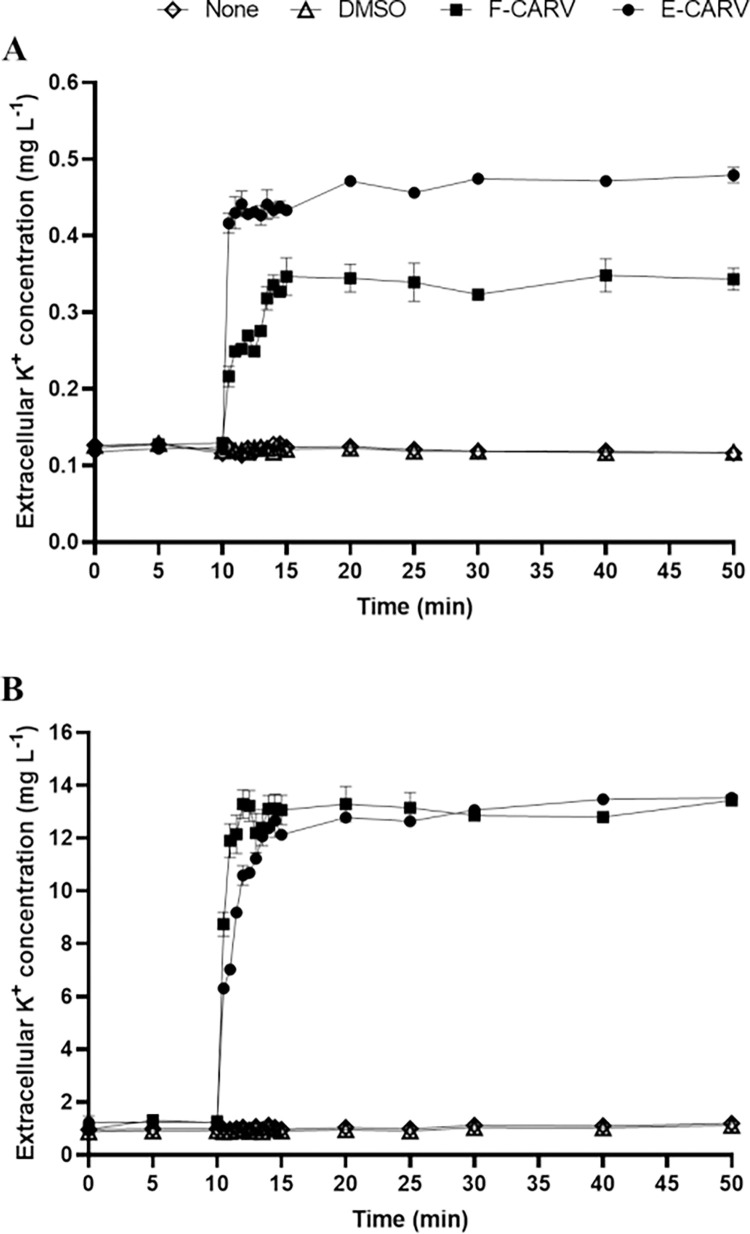
Kinetics of K^+^ ion leakage from the intracellular medium of *P*. *aeruginosa* (A) and *E*. *faecalis* (B) cells after treatment with F-CARV and E-CARV. The black arrow indicates the time of addition of F-CARV or E-CARV. The concentrations of K^+^ ions were measured using an atomic spectrometer and expressed as mg L^-1^. Data are presented as the means (±SD) of three independent experiments. Control represents cells treated with HEPES buffer containing DMSO for F-CARV and without DMSO for E-CARV.

In order to investigate the effect of carvacrol on bacterial membrane permeability, the extracellular K^+^ ions concentration was monitored. The results showed that after the addition of HEPES buffer (with or without DMSO), the concentration of extracellular K^+^ ions remained stable (Fig 5). However, after exposure of *P*. *aeruginosa* and *E*. *faecalis* cells to F-CARV and E-CARV at the MICs, the concentration of K^+^ ions in the extracellular medium of both bacterial suspensions increased immediately within 30 s of the treatment, and remained almost stable after 5 min of treatment. Forty minutes after exposure of *P*. *aeruginosa* cells to F-CARV (5 mg mL^-1^), the extracellular K^+^ concentration reached 0.34 mg L^-1^. This concentration was further increased (0.48 mg L^-1^) after treatment with E-CARV even using a 4-fold lower concentration (1.25 mg mL^-1^) (Fig 5A). However, for *E*. *faecalis*, the extracellular K^+^ concentration was similar (13.5 mg L^-1^) after forty minutes of treatment with F-CARV (0.625 mg mL^-1^) and E-CARV (0.625 mg mL^-1^) (Fig 5B).

#### Membrane disruption by carvacrol results in release of low-molecular-weight proteins

**Fig 6:**
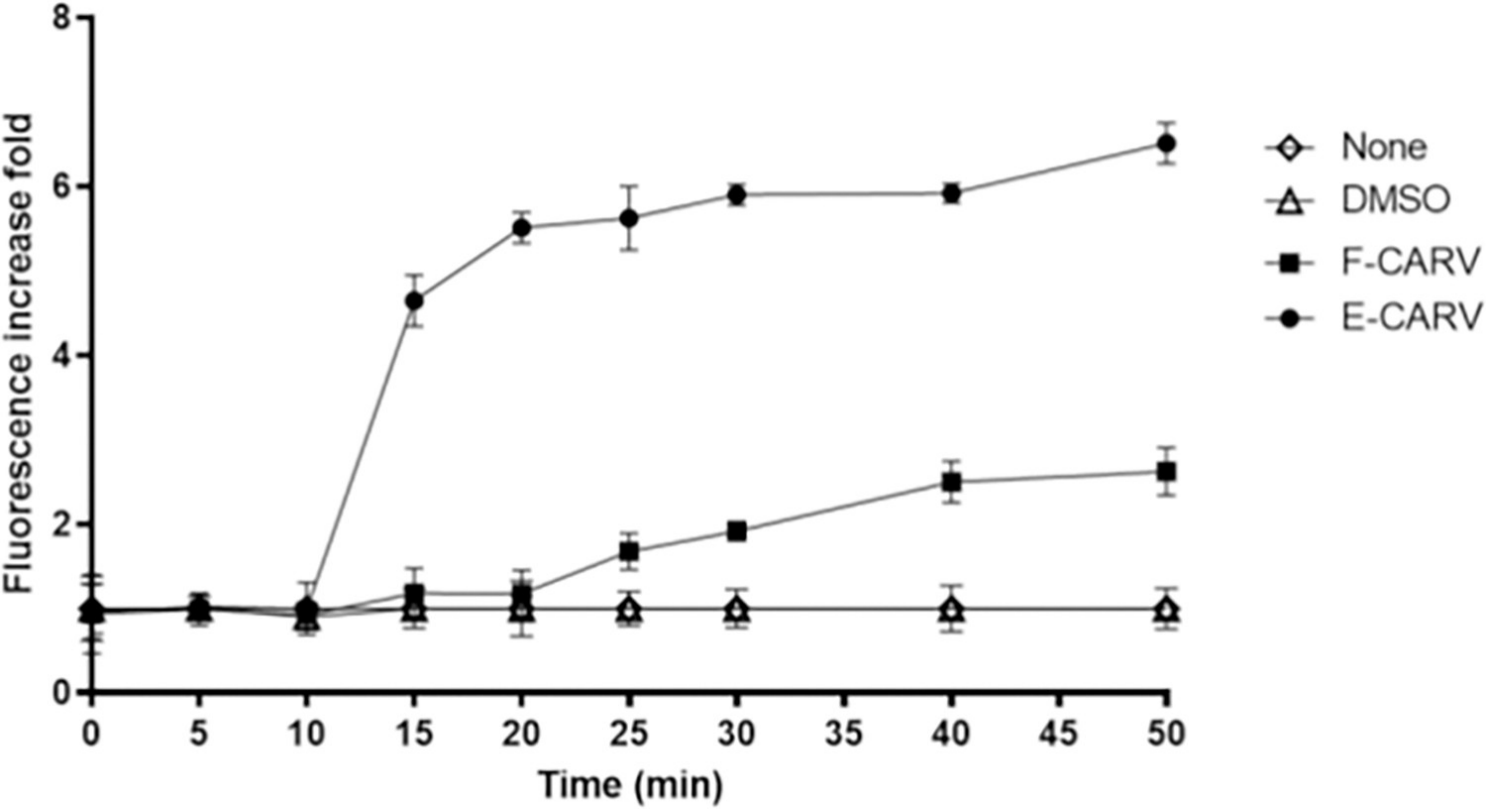
Assessment of cytoplasmic membrane permeability of *P*. *aeruginosa* GFP (ATCC ®10145GFP™) after treatment with F-CARV and E-CARV. The black arrow indicates the time at which F-CARV or E-CARV were added. The fluorescence of the GFP was measured using spectroscopy at an excitation and emission wavelength of 485 to 510 nm, respectively. Data are presented as means (±SD) of three independent experiments. Control represents cell treated with HEPES buffer containing DMSO for F-CARV and without DMSO for E-CARV.

To investigate the leakage of low-molecular-weight proteins, *P*. *aeruginosa* GFP strain was used. Fig 6 results showed that the negative control (with or without DMSO) had no effect on the extracellular fluorescence intensity of the bacterial population (Fig 6). By contrast, the GFP fluorescence intensity increased immediately after the addition of the carvacrol, demonstrating a significant instantaneous effect on membrane disruption (Fig 6). The results showed that this increase in GFP fluorescence intensity promoted by the treatment is time dependent. Indeed, after 5 min of bacterial treatment with F-CARV and E-CARV, the GFP fluorescence intensity increased by 1.1 and 4.6-fold compared to the control, respectively. While after forty minutes of exposure of bacteria to F-CARV, it increased by 2.6-fold, and this increase was significantly higher in intensity after E-CARV treatment and reached 6.5-fold (Fig 6).

#### Effect of carvacrol on *Pseudomonas aeruginosa* GFP cells viability

**Fig 7:**
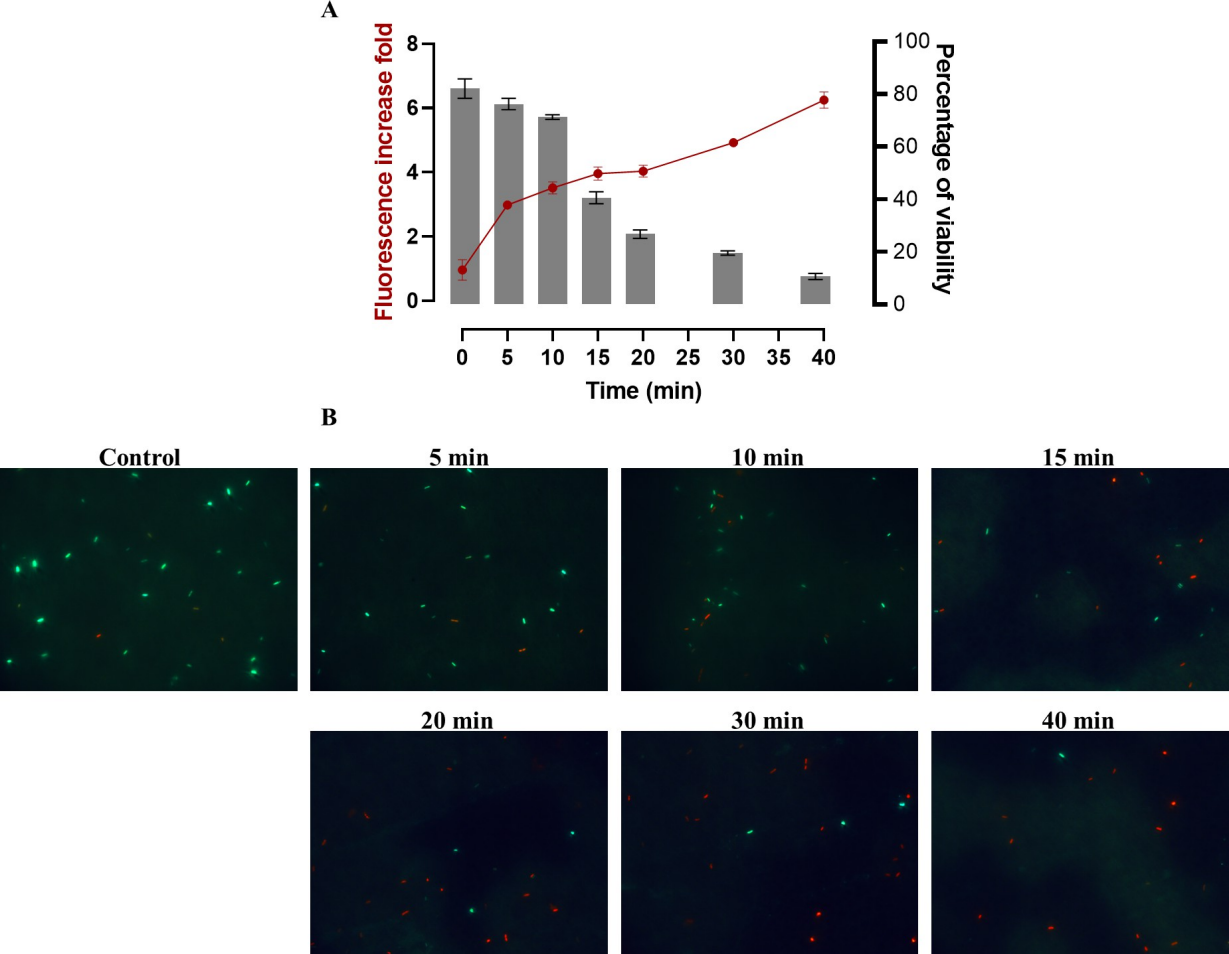
(A) Monitoring of extracellular GFP fluorescence intensity and cell viability of *P*. *aeruginosa* GFP over time after treatment with carvacrol at low concentration. (B) Epifluorescence microscopy visualization of *P*. *aeruginosa* GFP cells after staining with SYTO-9 (green fluorescence for living bacteria) and propidium iodide (red fluorescence for dead bacteria). Data are presented as the means (±SD) of three independent experiments. Control represents cells treated with HEPES buffer.

The number of viable cells as well as the intensity of GFP fluorescence were monitored after the exposure of *P*. *aeruginosa* GFP to a low concentration of the antimicrobial solution (0.2 mg mL^-1^). The results showed that the percentage of viable cells of *P*. *aeruginosa* GFP before treatment was 82% (Fig 7A). In addition, Fig 7B shows that the control harbored predominantly green cells (viable cells). However, treatment of bacteria with carvacrol was shown to progressively reduce the percentage of viable cells and increase the intensity of extracellular GFP fluorescence over time (Fig 7A). Indeed, after 15 min of treatment, the percentage of viable cells was reduced to 40% and the intensity of GFP fluorescence was increased by 3.9-fold. Furthermore, after 40 min of exposure of bacteria to carvacrol, the percentage of viable cells was further decreased to 11% and the extracellular GFP intensity was raised by 6.2-fold (Fig 7A). Similarly, Fig 7B shows that after treatment of bacterial cells with the antimicrobial solution, the number of green cells (viable cells) decreased and the number of red cells (dead cells) increased significantly over time. These results emphasize that the increase in extracellular GFP fluorescence intensity indicates cell death.

## Discussion

The use of natural plant-based substances, such as essential oils, has gained popularity as natural biocides alternative. This is due to their antimicrobial potential against a wide range of pathogenic bacteria whether in their planktonic or biofilm state, thus enabling the required treatment by overcoming the drawbacks of conventional antimicrobials used [27]. However, plant extracted compounds are generally not stable due to their volatility and vulnerability to photolysis and oxidation. In addition, the low solubility of these active compounds may reduce their antimicrobial efficacy [23,28,29]. It is therefore important to find the optimal formulation to go through these challenges. In our study, sodium caseinate was chosen as an emulsifier due to its ability to form an interfacial membrane around carvacrol droplets and supplemented with maltodextrins as wall material to achieve the best encapsulation efficiency.

SEM was used to assess the microstructure of spray-dried carvacrol microcapsules. Fig 1 showed that the microparticles had a spherical shape and bumpy surfaces. The shrinkage that occurred during the drying and cooling procedures might be the cause of some of the irregular surfaces which is a characteristic of the spray-drying process [30,31]. The micrographs of obtained microcapsules also showed that the surface indentation and roughness were more prominent in the small particles than in the large ones, suggesting that wall solidification occurred before the expansion of the microcapsules [32]. The study of the internal structure showed that carvacrol microcapsules were hollow, with a central void. The presence of air holes in the wall of microcapsules proved the presence of volatile compounds in the capsules. The voids might be the result of air expansion through spray drying process in the drops [33].

The MIC of F-CARV dissolved in DMSO against *P*. *aeruginosa* has shown a high value of 5 mg mL^-1^. As shown previously, the MIC of carvacrol against *P*. *aeruginosa* was higher than that of the other bacteria, which could be assigned to the resistance of *P*. *aeruginosa* associated with its efflux pump mechanism and β-lactamase activity [34,35]. However, results showed that E-CARV inhibited growth of bacteria at concentration 4-fold lower than those of F-CARV (1.25 mg mL^-1^). In addition, it was reported that the antimicrobial activity of carvacrol microencapsulated in hydroxypropyl-beta-cyclodextrin was higher than that of free carvacrol against both *Escherichia coli* and *Salmonella enterica* strains, indicating that encapsulation increased the water solubility and therefore the contact between carvacrol and bacteria in the medium [36]. Our results also showed that the MIC values of the F-CARV and E-CARV against *E*. *faecalis* were similar (0.625 mg mL^-1^). Although microencapsulation did not decrease the MIC value of carvacrol, the antimicrobial activity of carvacrol was retained under appropriate processing conditions. Other studies showed that, even F-CARV showed similar and even stronger antimicrobial activity compared to E-CARV. Nevertheless, the use of encapsulated carvacrol could be more interesting for food applications by masking the strong aroma of the compounds and ensuring a controlled release of carvacrol [37,38]. Furthermore, encapsulation is intended to protect the active compounds from environmental factors such as light, water, oxygen, pH, etc., thus maintaining their antibacterial action for a relatively longer period of time [39,40]. The time-kill assay is an appropriate and robust tool to gather information regarding the dynamic interaction between the bacterial strain and the antibacterial agent. Results showed that F-CARV and E-CARV exhibited a significant inhibition of viable bacterial cells for both tested strains compared to their controls, as well as a rapid bacterial reduction (1 and 5 min for *P*. *aeruginosa* and *E*. *faecalis*, respectively). However, other studies reported that carvacrol did not showed rapid activity using the MIC value, and that it required 6 h to reduce the bacterial population of *Salmonella* Typhimurium below the detection limit, even when exposed to the 2 MIC value, no viable cells were detected after 1 hour of exposure [41]. The relatively fast reduction in bacterial cell count is considered as important as the compound’s bactericidal nature, since more quickly the antimicrobial agent kills, more effectively it can inhibit the formation of biofilms [42]. The instantaneous bactericidal action of carvacrol observed in this study suggested that carvacrol may have affected the integrity of the bacterial membrane.

In addition, in order to study the effect of carvacrol against biofilm of bacterial strains, *NEC biofilm system* was used [25]. This system is a static biofilm system, which offers simplicity in the experimental procedure (biofilm formation and antibiofilm testing) and allows the study of the efficacy of disinfectants on biofilms. Furthermore, this system allows an easier access to the sessile cells, which permits the assessment of the biofilm formation and the disinfectant effectiveness through both cell counts and microscopic observations. The system also presents another advantage concerning its ability to receive all solid substrata, thereby enabling study of different solid surfaces [25]. In this study, stainless steel materiel was used as the solid surface to perform biofilms of *P*. *aeruginosa* and *E*. *faecalis*. Stainless steel is a material to which Gram-negative and Gram-positive bacteria, including *Pseudomonas* and *Enterococcus* strains, can adhere in a short time [43–45]. Our findings showed that after 24 h of incubation, biofilms of both bacteria exhibited a bacterial biomass of approximately 7 log CFU mL^-1^. Results showed that the applied carvacrol treatment resulted in a dose- and time-dependent reduction of total preformed biofilms. The biomass reduction of *P*. *aeruginosa* and *E*. *faecalis* biofilms after treatment with F-CARV and E-CARV was enhanced by increasing the time treatment as well as the carvacrol concentration (Fig 2). Thus, for both biofilms, the most effective treatment using F-CARV was obtained after 15 min treatment with MIC concentration that reduced biofilm biomass by approximately 5 log CFU mL^-1^ (*p < 0*.*05*). In addition, the biofilm reduction obtained using E-CARV after 15 min of treatment with MIC concentrations was 5.5 log CFU mL^-1^ for *E*. *faecalis*, and no bacterial counts were detected for *P*. *aeruginosa* (*p < 0*.*05*). Other studies showed that the use of higher concentrations of EOs result in severe cell membrane damage and a complete homeostasis disruption inducing cell death [41,46].

The increased efficacy of EOs with prolonged action time is due to a greater diffusion of the applied substances through the EPS matrix of the biofilm by prolonging the exposure time. This mode of action of EOs can be explained by the greater effectiveness of carvacrol after 15 min of exposure compared to a treatment with a short exposure time (1 min). Furthermore, our results showed that the antibacterial activity of E-CARV against *P*. *aeruginosa* and *E*. *faecalis* biofilm cells was significantly greater than that of F-CARV using either ½ MIC or MIC values. Hence, the encapsulation of carvacrol reduced the concentration of applied carvacrol while enhancing the anti-biofilm effect compared to the F-CARV.

These results are concordant with the biofilm analysis by epifluorescence microscopy, which allowed the direct observation of biofilms before and after treatment with F-CARV and E-CARV. Results showed that images of TS-treated (control) *P*. *aeruginosa* and *E*. *faecalis* biofilms stained with SYTO9 and PI showed compact biofilms composed predominantly of live bacteria (green cells) with minimal areas of dead bacterial cells (red cells) (Fig 4). However, after treatment with both F-CARV and E-CARV, the number of dead cells stained by PI increased. Moreover, treatment with E-CARV induced a higher percentage of dead cells compared to F-CARV. Thus, similar to previous results, this direct analysis provided evidence that treatment with the capsules showed significantly higher efficacy than un-encapsulated EOs, supporting the hypothesis that the capsules’ shell enhances the interaction with biofilms and induces effective penetration of the antimicrobials into the deep layers of biofilms [47,48].

Although various antimicrobial mechanisms have been described, bacterial cell walls and membranes are often considered the primary targets of EOs [49]. Thus, the effect of F-CARV and E-CARV treatment on cell membrane integrity was investigated by measuring the extracellular GFP fluorescence intensity and K+ ions concentration. The monitoring of the intensity of extracellular GFP fluorescence is a novel method developed by Khelissa et al. [24] who reported that the leakage of GFP to the extracellular medium is an indication of membrane permeabilization in stressed microbial cells. In this study, it was observed that exposure of *P*. *aeruginosa* and *E*. *faecalis* to F-CARV and E-CARV at the MICs increased the extracellular K^+^ ions concentrations immediately within 30 s of treatment (Fig 5). In addition, the extracellular GFP fluorescence intensity was gradually increased after both carvacrol treatment using *P*. *aeruginosa* GFP strain (Fig 6). This shows that F-CARV and E-CARV induced rapid release of potassium ions due to their small volume and progressive leakage of low-molecular-weight proteins from the cytoplasm over time because of their large volume. These results are in contradiction with our previous study, which showed that free and microencapsulated RUL3 did not induce protein leakage from the cytoplasm of *Escherichia coli* GFP (ATCC 25922GFP) [24]. The final extracellular GFP intensity after 40 min treatment of *P*. *aeruginosa* GFP with E-CARV was 3.9-fold higher than that measured after treatment with F-CARV, even at a concentration of E-CARV 4-fold lower than that of F-CARV (Fig 6). Thus, the results showed that microencapsulation enhances and reinforces the membrane disturbing action of carvacrol yet reducing the concentrations being used. Our finding also showed that the irreversible leakage of the intra-cellular bacterial pooled material leads to cell death (Fig 7). Hence, the results highlighted that carvacrol is able to target the cytoplasmic membrane of bacteria and disrupt the integrity of its phospholipid bilayer. Many previous studies have shown that the primary target of carvacrol is the bacterial membrane, as carvacrol is an inherently hydrophobic monoterpene that readily penetrates bacterial cell membranes, resulting in a disruption of their integrity and a subsequent release of bacterial cell contents and thus cell death [50–52]. In addition, SEM observations proved the damage to the cell membranes of both bacterial strains caused by carvacrol treatments (Fig 3). Our findings are in agreement with previous studies reporting that the spray-drying microencapsulation can be used as an effective tool to enhance the antibacterial agents’ activities while reducing the required concentrations [24,53].

## Conclusion

The present study investigated the microencapsulation of the volatile antibacterial agent carvacrol in a maltodextrin-sodium caseinate matrix using the spray-drying method. Our results suggested that the E-CARV exhibited a higher antimicrobial activity compared to F-CARV, while reducing the amounts of carvacrol required. The results obtained have shown a promising prospect of using microencapsulated carvacrol, as an alternative to conventional sanitizing methods to fight biofilms in the food industry and medical environments. However, before the application of these encapsulated products as disinfectants, they must be approved and registered by regulatory agencies. Finally, microencapsulation by spray-drying could be used to encapsulate other products in order to combat biofilms in the artificial ecosystems, and even to combat biofilms *in vivo*.

## Supporting information

S1 Graphical abstract (TIF)
