# Supplementary figures and images for: Microencapsulation of carvacrol as an efficient tool to fight Pseudomonas aeruginosa and Enterococcus faecalis biofilms
Source: PLoS One. 2022 Jul 1;17(7):e0270200. doi: 10.1371/journal.pone.0270200 (PMC9249205; doi:10.1371/journal.pone.0270200)

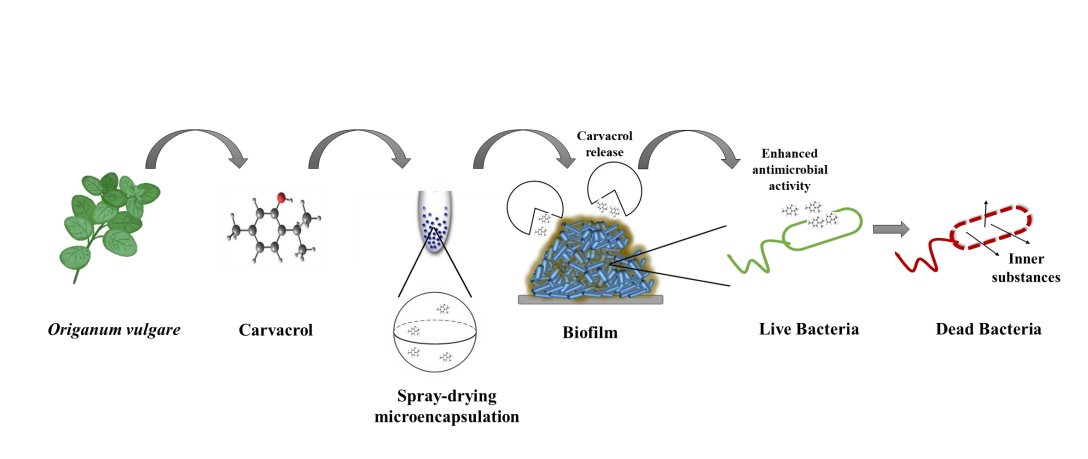

Supplement: S1 Graphical abstract — (TIF) [file pone.0270200.s001.tif]
